# Health worker training and day-1 feeding prescriptions for very low birth weight neonates: an interrupted time series study in two Kenyan hospitals

**DOI:** 10.3389/frhs.2026.1856111

**Published:** 2026-08-10

**Authors:** Allan Kayiza, Paul Laigong, Lucas Malla, Dalton Wamalwa, Grace Irimu

**Affiliations:** 1 Department of Paediatrics and Child Health, Kenya Methodist University, Meru, Kenya; 2 Department of Paediatrics and Child Health, University of Nairobi, Nairobi, Kenya; 3 London School of Hygiene and Tropical Medicine, London, United Kingdom

**Keywords:** very low birth weight, neonatal nutrition, health worker training, feeding prescriptions, interrupted time series, implementation science, quality improvement, Kenya

## Abstract

**Background:**

Optimal nutrition in very low birth weight (VLBW) neonates is essential in decreasing mortality and long-term morbidities but remains suboptimal in many newborn units (NBUs) in resource-limited settings. We assessed the effect of training healthcare practitioners (HCPs) on feeding prescriptions on the first day of life of VLBW neonates in two Kenyan newborn units.

**Methods:**

We conducted a quasi-experimental study using an interrupted time series (ITS) design. The intervention was HCP training on feeding of VLBW neonates. We extracted data on feed prescriptions issued on day one of life for all VLBW neonates admitted to the two newborn units. We calculated the proportion of correct feed prescriptions at different time points in the pre-interventional and post-interventional periods, and modelled changes using segmented beta regression.

**Results:**

We analyzed feed prescriptions for 181 and 254 neonates in the pre-intervention and post-intervention periods, respectively. The proportion of correct prescriptions increased from 38.1% to 45.3%, corresponding to an absolute effect (effect size) of 7.2 percentage points (95% CI −2.7–17.0). Segmented ITS analysis showed a declining pre-intervention trend in prescription correctness followed by an immediate step increase after the training. However, the improvement plateaued from the second month post-intervention. Neonatal stability, referral status, and place of admission were independently associated with incorrect feed prescriptions (*p* < 0.001, *p* = 0.008, and *p* = 0.007, respectively).

**Conclusions:**

A short, focused training intervention was associated with a modest, overall non-significant increase in the correctness of day-1 feeding prescriptions for VLBW neonates. ITS analysis suggested a possible early post-training improvement, but the segmented regression estimates were not statistically significant, and there was no evidence of a sustained effect over time. These findings suggest that training alone may be insufficient to achieve durable practice change without reinforcement and broader health system support.

## Background

Preterm birth remains a significant global challenge and a leading contributor to neonatal mortality. In Kenya, an estimated 11% of all deliveries are preterm, and complications of prematurity contribute to approximately 60%–80% of neonatal deaths, many of which are preventable through high-quality inpatient newborn care. Most of these deaths are linked to modifiable factors such as inadequate feeding, insufficient thermal care, and infections (1, 2).

Optimal nutrition is critical for survival and postnatal growth in preterm and very low birth weight (VLBW) neonates and reduces complications such as neonatal sepsis. Early initiation of enteral feeds promotes faster attainment of full feeds, shortens hospital stay, and improves outcomes (3, 4). In stable VLBW neonates, feeding should ideally begin within the first hour of life, using expressed breast milk (EBM) or donor breast milk (DBM) if EBM is unavailable. When parenteral support is required, careful attention to total fluid, energy, and protein prescription remains essential to ensure optimal growth (2, 5).

Standardized feeding protocols improve the nutritional management and outcomes of VLBW neonates. Kenya's national newborn care protocols recommend expressed breast milk at 80 mL/kg/day on day 1 for stable VLBW neonates. For unstable VLBW neonates, the protocols recommend intravenous 10% dextrose at 80 mL/kg/day, with trophic colostrum at 2 mL/kg every three hours after stabilization (6, 7). However, studies in Kenyan newborn units indicate poor adherence to these recommendations, with only about half of eligible neonates receiving appropriate feed volumes on day 1 (8). To address these gaps, Kenya's national newborn care guidelines emphasize standardized feeding practices and consistent implementation in newborn units (6, 7, 9).

Capacity-building in the application of these guidelines, together with skills reinforcement, represents a practical strategy for closing implementation gaps in busy, resource-limited newborn units. Previous studies have shown that short, focused training can improve clinical practices when aligned with clear protocols (10). However, the sustainability of training effects under routine conditions—and how performance changes over time—remains less well described.

We therefore evaluated the effect of a newborn feeding training package aligned with Kenya's national newborn care protocols, focusing on the correctness of feeding prescriptions on the first day of life for VLBW neonates in two Kenyan newborn units. Using a pragmatic interrupted time series (ITS) design, we assessed temporal changes in prescription correctness by examining pre-intervention trends, immediate post-training changes, and subsequent patterns over the follow-up period.

## Methods

We conducted a quasi-experimental study using an interrupted time series (ITS) design to evaluate the effect of newborn care training on the correctness of day-1 feeding prescriptions for very low birth weight (VLBW; 1,000–1,499 g) neonates.

The study was carried out in the newborn units of Pumwani Maternity Hospital (PMH), a county referral hospital admitting approximately 250 neonates monthly, and Kenyatta National Hospital (KNH), a national tertiary referral hospital admitting approximately 100 neonates monthly. Both hospitals participate in the Clinical Information Network (CIN), which supports standardized patient documentation, data-quality improvement, and routine audit and feedback (11).

The intervention consisted of training on the feeding of VLBW neonates, conducted at PMH in February 2021 and at KNH in March 2021. Training was delivered either through a five-day Comprehensive Newborn Care Training course or through a one-day focused session for staff who missed the main training (6, 7). Nearly all clinical staff and approximately 70% of nursing staff participated. The training included lectures, hands-on sessions, and simulation-based practice covering early enteral feeding of stable and unstable VLBW neonates, correct feed prescription using standardized charts, breast-milk expression, nasogastric or orogastric tube insertion and verification, and safe gastric-tube feeding. Family-centred care and infection prevention and control were emphasized throughout.

Data were extracted from the CIN REDCap database for the pre-intervention period from July 2020 to February 2021 and the post-intervention period from June 2021 to January 2022. CIN data clerks abstracted patient information from structured newborn admission forms and comprehensive vital-sign monitoring charts and entered the data into REDCap under supervision (11). Each ITS time point represented a four-week period. Eligible participants were neonates weighing 1,000 to <1,500 g who were admitted during the study period.

The primary outcome was the proportion of correct feeding prescriptions on day 1 of life. A prescription was classified as correct only if both the prescribed feed type and the prescribed total 24-hour feed volume were appropriate for the neonate's day-1 clinical status according to Section 1.5 of the Kenya Ministry of Health Comprehensive Newborn Care Protocols (7). For stable VLBW neonates, enteral feeds were the expected day-1 prescription, whereas for unstable VLBW neonates, intravenous fluids were the expected day-1 prescription. Unstable neonates were defined as those with convulsions, unconsciousness, severe respiratory distress with severe chest-wall indrawing, or absent bowel sounds. Correct volume was defined as a prescribed total 24-hour volume within ±20% of the guideline-recommended total daily volume. Detailed operational definitions and classification criteria are provided in Supplementary Tables S1, S2.

The minimum sample size was estimated at 36 VLBW neonates per time point using simulation methods for interrupted time series studies, assuming a 30% effect size and approximately 80% power (12). For the ITS analysis, the proportion of correct prescriptions at each time point was modelled using segmented beta regression to estimate changes in level and trend following the intervention. We also compared the overall difference between the pre-intervention and post-intervention periods using chi-square or Fisher's exact tests, as appropriate. All tests were two-sided, and statistical significance was set at *p* < 0.05.

Multivariable logistic regression with stepwise model selection based on Akaike's Information Criterion was used to identify factors associated with incorrect day-1 feeding prescriptions (13). Incorrect prescription was selected as the regression outcome to facilitate identification of characteristics associated with a higher likelihood of prescribing error.

Ethical approval was obtained from the University of Nairobi/Kenyatta National Hospital Ethics and Research Committee (P24/04/2021).

## Results

### Participant flow and baseline characteristics

**Figure 1:**
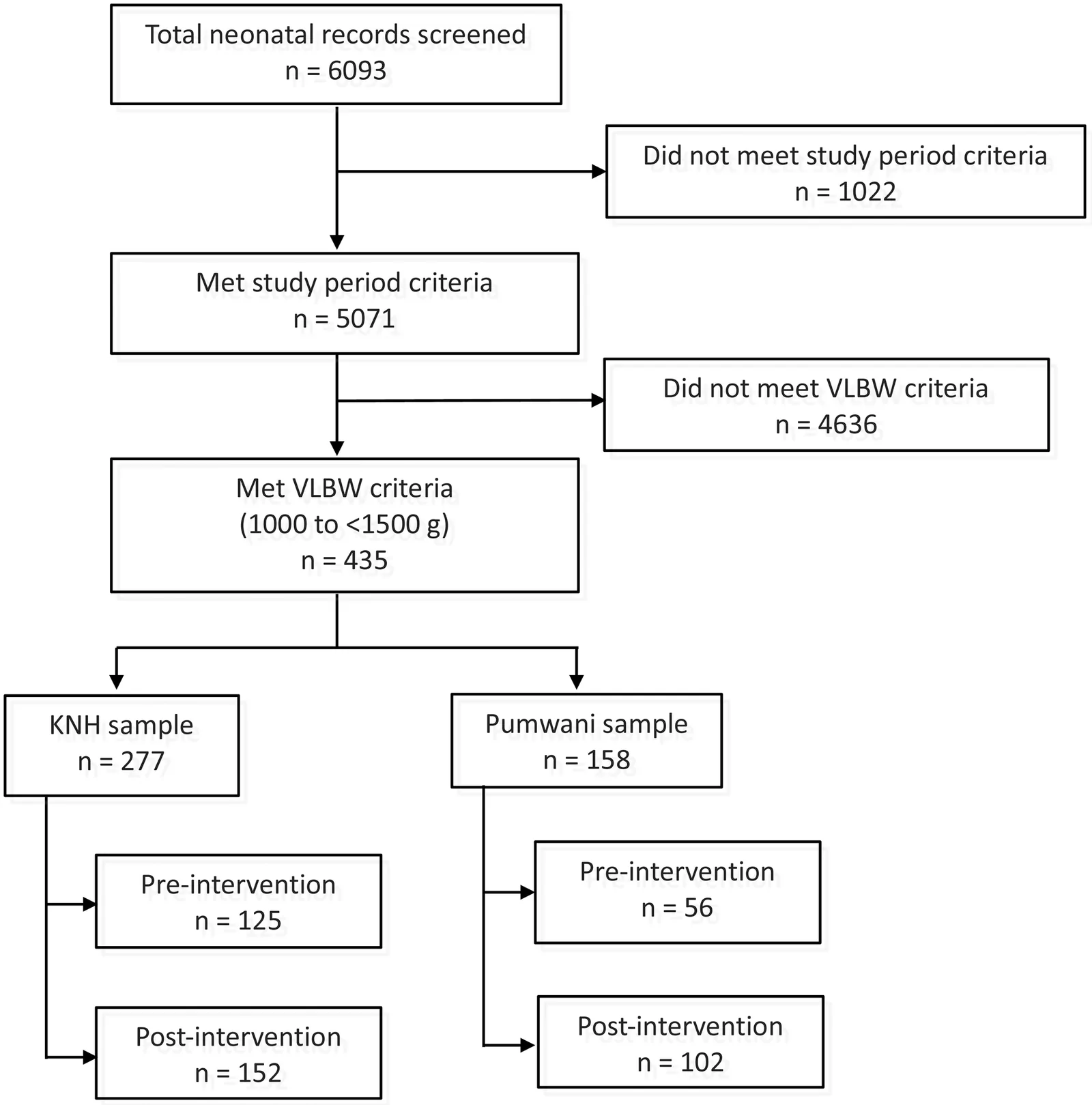
Flow diagram showing selection of VLBW neonates from the hospital database into the final analytic sample, by hospital and intervention period.

A total of 435 VLBW neonate records met the inclusion criteria, including 181 from the pre-intervention period and 254 from the post-intervention period. Of these, 277 were from KNH (125 pre-intervention and 152 post-intervention) and 158 were from PMH (56 pre-intervention and 102 post-intervention) (Figure 1).

Baseline demographic and clinical characteristics were broadly comparable between the pre- and post-intervention groups (Table 1). Females accounted for 53.0% (96/181) of the pre-intervention group and 45.7% (116/254) of the post-intervention group (*p* = 0.17). The proportion of neonates weighing 1,000–1,250 g was also similar in the two periods: 53.0% (96/181) vs. 50.0% (127/254) (*p* = 0.60). Gestational age was documented for 74% and 81.1% of neonates in the pre- and post-intervention periods, respectively; most documented neonates were born at 28–34 weeks’ gestation, with no significant difference between groups (*p* = 0.41). Maternal characteristics were not significantly different between the two periods. However, neonates in the post-intervention period were more likely to have been referred from another facility (26.0% vs. 17.7%; *p* = 0.04) and to have difficulty in breathing (73.6% vs. 64.6%; *p* = 0.018).

**Table 1:** Demographic and clinical characteristics of the study population.

Variable	Pre-intervention^a^		Post-intervention^a^		*P*-value^a^
Newborn characteristics	Frequency *N* = 181	Percent (%)	Frequency *N* = 254	Percent (%)	
Median birth weight (IQR)	1210gms (230)	_	1325gms (230)	_	0.51
Weight categories: 1,000–1,250 grams > 1,250 grams	96 85	53.0 47.0	127 127	50.0 50.0	0.60
Sex: Female Male Not documented	96 85 -	53.0 47.0 -	116 137 1	45.7 53.9 0.3	0.17
Gestational age at birth: 28–34 weeks > 34 weeks Not documented	120 14 47	66.3 7.7 26.0	191 15 48	75.2 5.9 18.9	0.41
Maternal characteristics					
Parity of the mother: Primigravida Multigravida	42 139	23.2 76.8	64 190	25.2 74.8	0.72
Age of the mother: <20 years 20–30 years 30–40 years >40 year	31 105 43 2	17.1 58.0 23.8 1.1	33 136 81 4	13.0 53.5 31.9 1.6	0.24
Newborn clinical characteristics					
HIV Sero-exposed: Yes No Not documented	8 153 20	4.3 84.5 11.2	13 210 31	5.1 82.7 12.2	0.89
Referral: Yes No Not documented	32 122 27	17.7 67.4 14.9	66 147 41	26.0 57.9 16.1	**0** **.** **04**
Difficulty in breathing: Yes No Not indicated	117 54 10	64.6 29.8 5.6	187 49 18	73.6 19.3 7.1	**0** **.** **018**
Mode of delivery: C-section Normal (SVD) Breech Not indicated	63 108 9 1	34.8 59.7 5.0 0.5	90 148 13 3	35.4 58.3 5.1 1.2	0.99
Had apnea: Yes No Not indicated	15 154 12	8.3 85.1 6.6	26 207 21	10.2 81.5 8.3	0.56
Had convulsions: Yes No Not indicated	1 158 22	0.6 87.3 12.3	2 231 21	0.8 90.9 8.3	1.00

Bold values indicate statistical significance at *P* < 0.05.

a Percentages are based on column totals. *P*-values were calculated using chi-square or Fisher's exact test as appropriate, excluding records with missing data for the variable under comparison.

### Effect of the training on feed prescription correctness

Overall, the proportion of VLBW neonates receiving a correct day-1 feed prescription increased from 38.1% (69/181) in the pre-intervention period to 45.3% (115/254) in the post-intervention period. This represented an absolute increase of 7.2 percentage points (95% CI −2.7–17.0), but the difference was not statistically significant (*p* = 0.14). There was also no significant difference between the two periods in the individual components of prescription correctness, namely correct 24-hour feed volume and correct feed type (Table 2).

**Table 2:** Comparison of feeding prescription indicators in the pre-intervention and post-intervention periods.

Indicator	Pre-intervention	Post-intervention	*P*-value	Effect size% (95% CI)
All study neonates				
Correct feed prescription (correct volume and type)	69/181 (38.1%)	115/254 (45.3%)	0.14	+7.2% (−2.7 to 17.0)
Correct 24-hr volume of feeds:	101/181 (55.8%)	143/254 (56.3%)	1.00	**+0.5% (−9.4 to 10.4)**
Correct type of feed	94/181 (51.9%)	138/254 (54.3%)	0.69	**+2.4% (−7.5 to 12.4)**
Subset analysis by neonatal stability				
Unstable neonates				
Prescribed intravenous fluids without enteral feeds	88/119 (74%)	131/188 (69.7%)	0.50	−4.3% (−15.2 to 6.6)
Inappropriately prescribed enteral feeds	15/119 (12.6%)	19/188 (10.1%)	0.69	−2.5% (−7.6 to 12.3)
Stable neonates				
Appropriately prescribed enteral feeds	21/62 (33.9%)	14/66 (21.2%)	0.16	−12.7% (−29.6 to 4.2)
Inappropriately prescribed intravenous fluids	45/62 (72.6%)	36/66 (54.5%)	**0** **.** **03**	−18.1% (−34.4 to −1.7)

Note: Trophic feeds not included. Stable VLBW neonates were expected to receive enteral feeds on day 1 of life, whereas unstable VLBW neonates were expected to receive intravenous fluids without enteral feeds. Correct feed prescription was defined as prescription of both the appropriate 24-hour volume and the appropriate feed type according to Ministry of Health guidelines.

Bold values indicate statistical significance at *P* < 0.05.

Among unstable VLBW neonates, the proportion appropriately prescribed intravenous fluids without enteral feeds in the first 24 h decreased slightly from 74.0% (88/119) to 69.7% (131/188), an absolute difference of −4.3 percentage points (*p* = 0.50). The proportion of unstable neonates inappropriately prescribed enteral feeds on day 1 also decreased, from 12.6% (15/119) to 10.1% (19/188), an absolute difference of −2.5 percentage points. These changes were not statistically significant (*p* = 0.69).

Among stable VLBW neonates, for whom enteral feeds were the recommended day-1 prescription, the proportion appropriately prescribed enteral feeds decreased from 33.9% (21/62) before the intervention to 21.2% (14/66) after the intervention, an absolute difference of −12.7 percentage points (95% CI −29.6–4.2; *p* = 0.16). The proportion inappropriately prescribed intravenous fluids also decreased, from 72.6% (45/62) to 54.5% (36/66), an absolute difference of −18.1 percentage points (95% CI −34.4 to −1.7; *p* = 0.03) (Table 2).

### Interrupted time series (ITS) analysis

**Figure 2:**
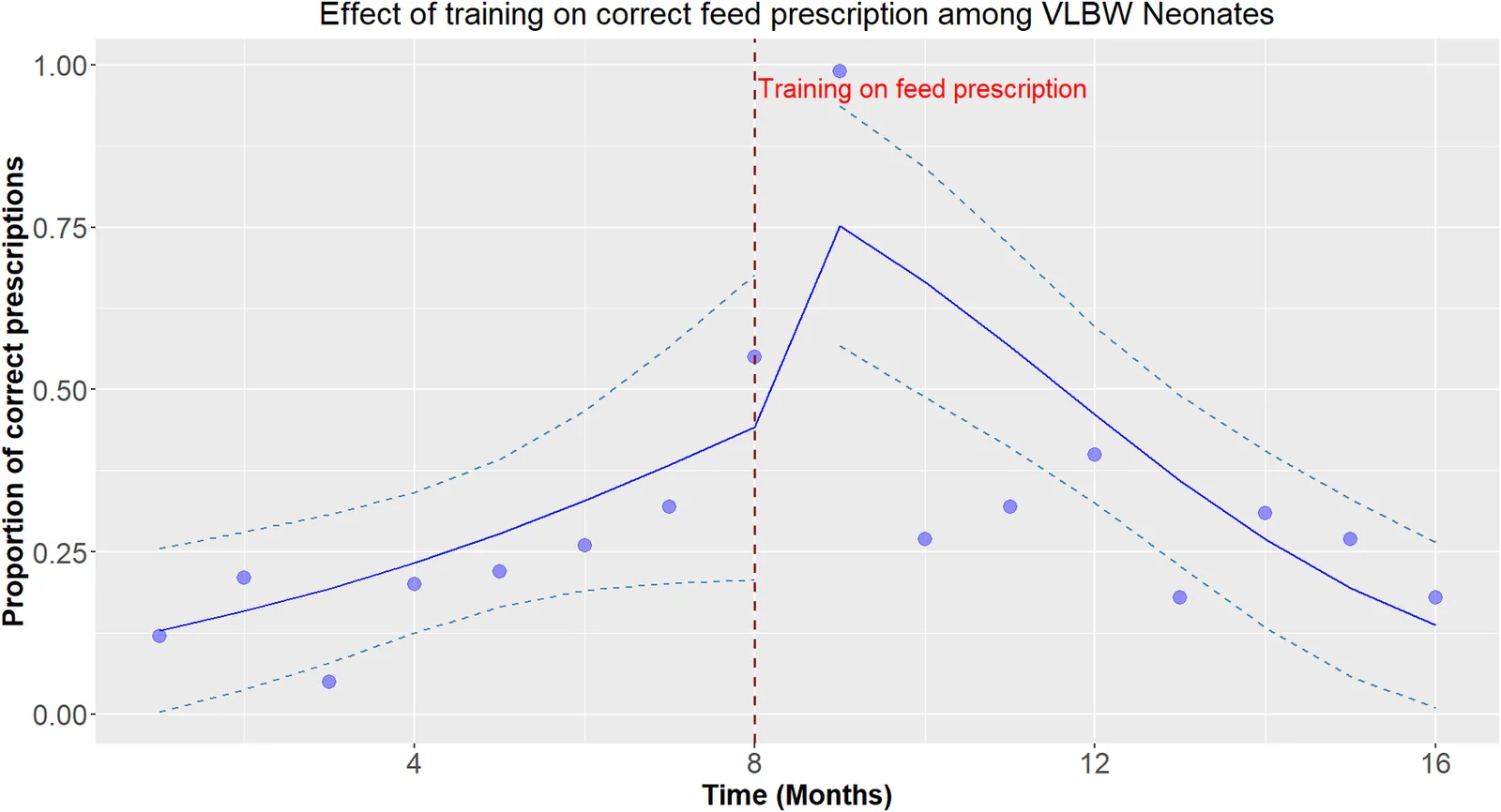
Interrupted time series plot showing changes in the proportion of correct Day-1 feeding prescriptions before and after training.

The ITS analysis showed that the proportion of correct feed prescriptions declined during the pre-intervention period. Following implementation of the training, there was an immediate increase in the prescription correctness, indicating a possible short-term intervention effect. However, this improvement was not sustained, and performance appeared to plateau from approximately the second month after training (Figure 2).

In the segmented regression model, the pre-intervention trend was negative but not statistically significant (*β* = −0.10, 95% CI −0.82–0.56; *p* = 0.78), indicating no statistically significant change in prescription correctness during the pre-intervention period. The immediate level change following training was positive but not statistically significant (*β* = 0.80, 95% CI −3.22–5.21; *p* = 0.70). The post-intervention slope change was also positive, small, and non-significant (*β* = 0.10, 95% CI −0.80–1.02; *p* = 0.82), providing no evidence of a sustained improvement over time. Overall, the findings suggest a possible early post-training change, but no statistically significant immediate or sustained intervention effect was demonstrated (Table 3).

**Table 3:** Segmented regression analysis of interrupted time series changes in correct feed prescription before and after training.

Parameter	Estimate (*β*)	95% CI	*P* – value
Baseline trend	−0.10	−0.82 to 0.56	0.78
Immediate level change	0.80	−3.22 to 5.21	0.70
Post-intervention slope change	0.10	−0.80 to 1.02	0.82

Note: β, regression coefficient; CI, confidence interval. The segmented regression model estimated the baseline level (intercept), pre-intervention time trend, immediate level change following training, and change in trend during the post-intervention period. Positive coefficients indicate an increase in the proportion of correct feed prescriptions, whereas negative coefficients indicate a decrease.

### Factors associated with incorrect day-1 feeding prescription

As no statistically significant effect of training on prescription correctness was observed, data from the pre-intervention and post-intervention periods were pooled for bivariate analysis. Only neonatal stability and referral status were significantly associated with prescription correctness. Unstable neonates had substantially lower odds of receiving an incorrect prescription than stable neonates (OR 0.02, 95% CI 0.008–0.06; *p* < 0.001). Conversely, referred neonates had more than twice the odds of receiving an incorrect prescription compared with neonates born within the study facilities (OR 2.28, 95% CI 1.38–3.78; *p* = 0.001). No significant associations were observed for sex, place of admission, mode of delivery, or gestational age (Table 4).

**Table 4:** Patient factors associated with day-1 feed prescription correctness among VLBW neonates.

Variable	Prescriptions			
Variable	Correct *n* (%)	Incorrect *n* (%)	Crude OR (95% CI) of incorrect prescription	*p*-value
Sex Female (ref) Male	86 (40.4%) 98 (44.1%)	127 (59.6%) 124 (55.9%)	0.86 (0.59, 1.25)	0.43
Place of admission KNH (Ref) Pumwani	119 (52.4%) 65 (41.1%)	158 (47.6%) 93 (58.9%)	1.1 (0.73, 1.60)	0.71
Neonatal stability Stable (ref) Unstable	4 (3.1%) 180 (58.6%)	124 (96.9%) 127 (41.4%)	0.02 (0.008, 0.06)	<0.001
Referral status^a^: No (ref) Yes	125 (46.5%) 27 (27.6%)	144 (53.5%) 71 (72.4%)	2.28 (1.38, 3.78)	0.001
Mode of delivery^a^: Caesarian section (ref) Vaginal delivery	67 (43.8%) 108 (42.2%)	86 (56.2%) 148 (57.8%)	1.07 (0.71, 1.60)	0.75
Gestational age^a^: 28–34 weeks (ref) >34 weeks	142 (45.7%) 9 (31%)	169 (54.3%) 20 (69%)	1.87 (0.82, 4.23)	0.13

a Referral status, mode of delivery, and gestational age had missing data; therefore, denominators varied across variables.

### Multivariable analysis

Multivariable logistic regression identified neonatal stability, referral status, and place of admission as independent factors associated with incorrect day-1 feeding prescriptions. After adjustment for the other variables in the model, unstable neonates had markedly lower odds of receiving an incorrect prescription than stable neonates (adjusted OR 0.02, 95% CI 0.006–0.056; *p* < 0.001). Referred neonates had more than twice the odds of incorrect prescription compared with neonates who were not referred (adjusted OR 2.20, 95% CI 1.23–3.98; *p* = 0.008). In addition, admission at PMH was independently associated with higher odds of incorrect prescription compared with admission at KNH (adjusted OR 2.13, 95% CI 1.23–3.72; *p* = 0.007) (Table 5).

**Table 5:** Multivariable analysis of factors associated with incorrect day-1 feed prescription among VLBW neonates.

Variable	Prescriptions			
	Correct n (%)	Incorrect *n* (%)	Adjusted OR (95% CI) of incorrect prescription*	*p*-value
Neonatal stability Stable (ref) Unstable	4 (3.1%) 180 (58.6%)	124 (96.9%) 127 (41.4%)	0.02 (0.006, 0.056)	<0.001
Referral status No (ref) Yes	125 (46.5%) 27 (27.6%)	144 (53.5%) 71 (72.4%)	2.20 (1.23, 3.98)	0.008
Place of admission KNH (ref) Pumwani	119 (52.4%) 65 (41.1%)	158 (47.6%) 93 (58.9%)	2.13 (1.23, 3.72)	0.007

* Adjusted odds ratios represent the odds of incorrect feed prescription relative to the reference category.

## Discussion

This study evaluated the effect of a focused newborn feeding training intervention on day-1 feeding prescription practices for VLBW neonates in two Kenyan newborn units. Overall, correct prescriptions increased from 38.1% before the intervention to 45.3% after the intervention, representing a modest absolute improvement that was not statistically significant. These findings suggest that although training may contribute to improved prescribing practices, the observed overall improvement under routine service conditions was limited.

The interrupted time series analysis provided additional context beyond the overall pre–post comparison. It showed a declining pre-intervention pattern, followed by an apparent immediate increase in prescription correctness after training, with subsequent plateauing from about the second month after the intervention. However, the segmented regression estimates were not statistically significant, and these temporal patterns should therefore be interpreted cautiously. Rather than demonstrating a confirmed intervention effect, the ITS findings suggest a possible short-term improvement, with no evidence of sustained improvement over time. This interpretation is consistent with broader literature showing that focused training may produce early improvements in health-worker knowledge and clinical practice (10, 14–16), but that sustained practice may require reinforcement through strategies such as audit and feedback, supportive supervision, mentorship, and other system-level supports (11, 17–19).

An important and somewhat concerning finding was observed among stable VLBW neonates. Although enteral feeding was the recommended day-1 prescription for these neonates, the proportion appropriately prescribed enteral feeds decreased from 33.9% before the intervention to 21.2% after the intervention, although this change was not statistically significant. In contrast, inappropriate prescription of intravenous fluids decreased significantly from 72.6% to 54.5%. This mixed pattern suggests that one form of inappropriate prescribing decreased after training, but recommended enteral feeding practice among stable VLBW neonates did not improve. Early enteral feeding is recommended for clinically stable VLBW neonates and has been associated with earlier attainment of full feeds and improved nutritional management (3, 4, 6, 7). One possible explanation for the observed findings is that health workers remained hesitant to initiate enteral feeds because of perceived concerns about feeding complications. However, these motivations were not measured in this study. In routine-care settings, adherence to protocol recommendations may require continued implementation support, including audit and feedback, supervision, and mentorship (11, 17–19).

Among unstable VLBW neonates, the proportion appropriately prescribed intravenous fluids without enteral feeds remained relatively high in both periods, with small, non-significant decreases after training. Similarly, inappropriate enteral feeding decreased slightly, but the change was not statistically significant. These findings suggest that the intervention did not materially change day-1 prescribing practices among unstable neonates. Taken together, the subgroup findings suggest that factors beyond knowledge of guidelines may influence feeding decisions in VLBW neonates; however, these factors were not directly assessed in this study.

The multivariable analysis also provides insight into where prescribing challenges may be greatest. Unstable neonates had substantially lower odds of receiving an incorrect prescription than stable neonates, while referred neonates and those admitted at PMH had higher odds of receiving an incorrect prescription. These findings suggest that prescribing behaviour may vary according to neonatal stability, referral status, and facility context. Referred neonates may present with greater clinical uncertainty or incomplete information, while facility-level differences may reflect variation in workflow, staffing, supervision, and uptake of training content. These contextual influences reinforce the importance of viewing prescribing quality as both a clinical and a health-system issue. These explanations were not directly evaluated and should be regarded as hypotheses for future investigation.

Taken together, the findings point to the limitations of training as a stand-alone strategy. Focused in-service training remains important, particularly for introducing or standardizing evidence-based care, but it is unlikely to be sufficient on its own to produce durable behaviour change. Sustaining improvement over time will likely require training to be embedded within broader quality-improvement and implementation strategies, including refresher sessions, supportive supervision, clinical mentorship, routine audit and feedback, and practical decision-support tools integrated into daily care (11, 17, 18). Adoption, fidelity, and sustainability are distinct implementation outcomes and may require different forms of support (19).

These findings should be interpreted in light of several limitations, including the non-significant overall and segmented ITS effects, reliance on routinely collected clinical data, and the fact that the analysis assessed prescription correctness rather than actual feed delivery or neonatal outcomes. The study also did not formally identify or control for other influences on healthcare workers’ knowledge or prescribing practices during the study period, such as concurrent training, informal mentorship, staff turnover, or other quality-improvement activities. Therefore, the possibility that factors other than the study intervention contributed to the observed changes cannot be excluded.

## Conclusion

In summary, short, focused newborn care training was associated with a modest, overall non-significant improvement in day-1 feed prescription practices for VLBW neonates. The interrupted time series analysis suggested a possible early post-training improvement, but there was no evidence of a sustained effect over time. Among stable VLBW neonates, inappropriate intravenous-fluid prescribing decreased after the intervention, although recommended enteral feeding did not improve. These findings suggest that training alone may be insufficient to achieve durable and comprehensive improvements in prescribing practice. Embedding training within broader, multifaceted quality-improvement strategies may be necessary to support sustained adherence to feeding guidelines and improve neonatal care.

## Recommendations

Regular, short, focused in-service training on feeding VLBW neonates should be considered as one component of efforts to improve prescription accuracy in newborn units. However, training should be reinforced through additional evidence-based strategies such as refresher sessions, audit and feedback, supportive supervision, and clinical mentorship to improve and sustain adherence to national and WHO feeding guidelines. Future research should determine the optimal interval for refresher training and assess the effectiveness of multifaceted implementation strategies in improving both prescribing practice and neonatal outcomes.

## Data Availability

The datasets presented in this study can be found in online repositories. The names of the repository/repositories and accession number(s) can be found in the article/Supplementary Material.
