# Supplementary material for: Health worker training and day-1 feeding prescriptions for very low birth weight neonates: an interrupted time series study in two Kenyan hospitals
Source: Front Health Serv. 2026 Aug 10;6:1856111. doi: 10.3389/frhs.2026.1856111 (PMC13500493; doi:10.3389/frhs.2026.1856111)
Supplement: Supplementary file 1 [file Datasheet1.pdf]

SUPPLEMENTARY TABLES

Study: Health worker training and day-1 feeding prescriptions for very low birth weight neonates: An interrupted time series study in two Kenyan hospitals

Supplementary Table S1: Operational definition and classification criteria for day 1 feed prescription correctness among VLBW neonates

**Component**	**Stable VLBW neonate**	**Unstable VLBW neonate**
Clinical definition used for classification*	Neonate without convulsions, unconsciousness, severe respiratory distress/severe chest wall indrawing, or absent bowel sounds.	Neonate with any of the following: convulsions, unconsciousness, severe respiratory distress/severe chest wall indrawing, or absent bowel sounds.
Guideline source	Kenya Ministry of Health, Comprehensive Newborn Care Protocols (November 2022), section 1.5a: Feeding stable and well babies – birth weight less than 1.5 kg.	Kenya Ministry of Health, Comprehensive Newborn Care Protocols (November 2022), section 1.5b: Feeding unstable babies.
Expected feed type on day 1	Enteral feeds using expressed breast milk (EBM), usually via NGT/OGT for babies 1000–1499 g.	Intravenous fluids (10% dextrose) for the first 24 hours; trophic colostrum may be given after stabilization according to the guideline but was not included in the primary feed type classification
Day 1 volume standard used in this study	Total enteral feed volume appropriate for birth weight and day 1, based on guideline tables for stable newborns less than 1.5 kg.	Total IV fluid volume appropriate for birth weight and day 1, based on guideline tables for unstable newborns less than 1.5 kg.
Volume classified as correct if	Prescribed total 24 hour volume was within ±20% of the guideline-recommended total daily volume.	Prescribed total 24 hour volume was within ±20% of the guideline-recommended total daily volume.
Feed type classified as correct if	Prescription specified enteral feeds rather than IV fluids alone.	Prescription specified IV fluids as the primary day 1 feeding approach
Overall prescription classified as correct if	Both feed type and total 24 hour volume were correct.	Both feed type and total 24 hour volume were correct.
Classification as incorrect if	Either wrong feed type, wrong volume, or both.	Either wrong feed type, wrong volume, or both.

**Classification was based on the Kenya Ministry of Health Comprehensive Newborn Care Protocols, section 1.5, including the clinical pathway for stable newborns less than 1.5 kg and the feeding/fluid pathway for unstable newborns. Unstable newborn characteristics were defined by the protocol as convulsions, unconsciousness, severe respiratory distress with severe chest wall indrawing, or absent bowel sounds.*

Supplementary Table S2: Guideline based day-1 feed volumes used to assess prescription correctness in VLBW neonates (1000–1499 g)

**Birth weight**	**Stable VLBW neonate**		**Unstable VLBW neonate**	
	Recommended day 1 EBM total volume	Approximate 3 hourly feed volume	Recommended day 1 IV fluid total volume	Trophic colostrum
1.0 kg	80 ml/kg/day	10 ml every 3 hours	80 ml/kg/day	2 ml/kg every 3 hours after stabilization
1.1 kg	80 ml/kg/day	11 ml every 3 hours	80 ml/kg/day	2 ml/kg every 3 hours after stabilization
1.2 kg	80 ml/kg/day	12 ml every 3 hours	80 ml/kg/day	2 ml/kg every 3 hours after stabilization
1.3 kg	80 ml/kg/day	13 ml every 3 hours	80 ml/kg/day	2 ml/kg every 3 hours after stabilization
1.4 kg	80 ml/kg/day	14 ml every 3 hours	80 ml/kg/day	2 ml/kg every 3 hours after stabilization

**For stable babies with birth weight less than 1.5 kg, the Kenya Ministry of Health protocol recommends starting EBM at 80 ml/kg/day on day 1 and increasing by 20 ml/kg/day thereafter. For unstable newborns with birth weight less than 1.5 kg, the protocol recommends IV 10% dextrose at 80 ml/kg/day on day 1, with trophic colostrum 2 ml/kg every 3 hours after stabilization; trophic feeds are not deducted from IV fluids.*

Supplementary Methods S1. Interrupted time series structure and outcome classification

The interrupted time series (ITS) analysis was conducted using aggregated four-week time points. Data for the pre-intervention period were extracted from July 2020 to February 2021, and data for the post-intervention period from June 2021 to January 2022. The intervention consisted of training on feeding of VLBW neonates, delivered at Pumwani Maternity Hospital in February 2021 and at Kenyatta National Hospital in March 2021**.** Each ITS time point represented the proportion of eligible VLBW neonates with a correct day-1 feed prescription during a four-week period.

The primary outcome was the proportion of correct feeding prescriptions on day 1 of life. A prescription was classified as correct only if both the prescribed feed type and the prescribed total 24 hour feed volume were appropriate for the neonate's day-1 clinical status according to the Kenya Ministry of Health Comprehensive Newborn Care Protocols (November 2022), section 1.5 Feeds and Fluids. For stable VLBW neonates, enteral feeding with expressed breast milk was the expected day-1 prescription. For unstable VLBW neonates, intravenous fluids were the expected day-1 prescription; the protocol defines unstable newborns as those with convulsions, unconsciousness, severe respiratory distress with severe chest wall indrawing, or absent bowel sounds**.** Correct volume was defined as a prescribed total 24-hour volume within ±20% of the guideline-recommended total daily volume.

For stable newborns with birth weight less than 1.5 kg, the protocol recommends EBM 80 ml/kg/day on day 1**,** increased by 20 ml/kg/day thereafter, with weight-based three-hourly feed volumes provided for newborns weighing 1000 – 1499gms**.** For unstable newborns with birth weight less than 1.5 kg, the protocol recommends IV 10% dextrose at 80 ml/kg/day on day 1, with 2 ml/kg colostrum every 3 hours after stabilization as trophic feeds, not deducted from the IV fluid prescription. These protocol rules formed the basis for the supplementary coding tables used in classifying prescriptions as correct or incorrect.
